# Immunohistochemical Results of Soft Tissues Around a New Implant Healing-Abutment Surface: A Human Study

**DOI:** 10.3390/jcm9041009

**Published:** 2020-04-02

**Authors:** Barbara Ghinassi, Gianmaria D’Addazio, Angela Di Baldassarre, Beatrice Femminella, Giorgio Di Vincenzo, Maurizio Piattelli, Giulia Gaggi, Bruna Sinjari

**Affiliations:** 1 Department of Medicine and Aging Sciences, University "G.d’Annunzio" of Chieti-Pescara, 66100 Chieti, Italy; b.ghinassi@unich.it (B.G.); angela.dibaldassarre@unich.it (A.D.B.); giulia.gaggi@unich.it (G.G.); 2 Department of Medical, Oral and Biotechnological Sciences, University “G. d’Annunzio” of Chieti-Pescara, 66100 Chieti, Italy; gianmariad@gmail.com (G.D.); beatrice.femminella@yahoo.it (B.F.); maurizio.piattelli@unich.it (M.P.); 3 Department of Periodontics & Implant Dentistry, New York University, E 40th St #508, New York, NY 10016, USA; gtd2@nyu.edu

**Keywords:** dental implant, implant surface, peri-implant soft tissues, laser treated, immunohistochemical analysis

## Abstract

Although, the high success rate of implant rehabilitation treatment, the biological complications such as bone loss and peri-implantitis are still present. The creation of a coronal biological seal between the implant and the oral tissues seems to be a crucial point on preserving dental implants. The objective of this study was to immunohistochemically analyze the behavior of peri-implant soft tissues around a new implant healing-abutment surface on humans. A total of 30 soft tissue biopsies were collected after a healing period of 30 (±7) days, to analyze the expression of inflammatory (cluster of differentiation 63 (CD63), human neutrophil peptides 1–3 (HPN1–3)) and junctional (E-cadherin, occludin, and β-catenin) markers, on soft tissues around laser treated and machined alternated healing abutments. The evaluation demonstrated the whole area of the soft tissues adherent to the laser treated surface with a regular morphology. While several stress hallmarks in correspondence of machined surfaces were shown such as: (a) An irregular, disrupted, and discontinued basal membrane with an increased inflammation evident both the epithelial and connective tissues; (b) the absence or defective proper keratinization process of the external layer, and (c) damages in the cell to cell interaction. In conclusion, the laser treated surface is preferable to maintain the integrity and functionality of the gingiva epithelium.

## 1. Introduction

Over the years, implant-prosthetic rehabilitations have shown to have high predictability and long-term follow-ups, being a valid solution for the substitution of missing teeth [1]. The early success rate has been confirmed around 94.6%, meanwhile after 10 years of function they decrease to 89.7% after more than [1,2]. Different factors have been analyzed over the years to optimize the clinical procedures influencing the follow-up of the restorations and possible complications [3,4]. Among these factros, fixture abutment interface, marginal bone behavior, and bacterial leakage represent some of the trending topics in the literature to study the implants’ behavior [5,6,7]. Complications and implant failure necessitate new surgical interventions, repair or substitution causing, additionally, considerable economic and healthcare drawbacks [8]. One of the most important biological complications is the bacterial load around dental implants which leads to bone resorption and/or peri-implantitis [9]. The latter has been described as a destructive inflammatory lesion affecting both hard and soft tissues causing bone loss and implants failure [9]. A recent report showed a prevalence of peri-implantitis of 36.6% at 8.4 years of loading [10]. Regardless of being a multifactorial disease, different studies have analyzed the relationship between biofilm and bone loss and searched for the influence of abutment materials physical-chemical properties related to implant success/failure [11]. A crucial point on this regard plays the implant fixture/abutment gap formation on a two-piece dental implant [12]. In fact, it is almost recognized that a coronal biological seal is crucial for preventing microbial colonization. Another aspect, although still being studied, is related to the establishment of this soft tissue barrier. It appears a key part of tissue integration being the result of wound healing established at the interface between living tissues and a foreign body [9,13]. Disorders in the structure of epithelial gingiva, with main regards to cell to cell interactions, impairs the defense against periodontopathogens and increase the possibility that other less pathogens bacteria, such as *Fusobacterium nucleatum* and *Prevotella intermedia*, can invade the tissue [14]. It has been shown that the epithelium and cell junctions play an important role against bacterial invasion [15], but also neutrophils play a pivotal role in host defense and their number increase with the periodontal disease progression and the bacteria plaque growth. The granules of neutrophils contain many antibacterial molecules such as lipocalin, lysozyme and small peptides. Among the antimicrobial peptides there are the human neutrophil defensins (HNPs) that contribute to bacteria killing. It has been shown that HNPs increases during periodontal disease affecting the healthy periodontium epithelial cells. HNP1–3, in particular, acts as chemoattractant for T-cells and monocytes and its high concentration induces epithelial cells death and stimulates bacterial adhesion to the epithelium [14].

On the other hand, numerous adhesion molecules have been studied with the aim to better understand the biological behavior and role to the adhesion of dental or peri-implant soft tissues. Among these molecules we can find the: E-cadherin which has a prominent partin the development of tight junctions [16,17]; β- catenin, a cytoplasmic and nuclear protein able to bind the cytoplasmatic domain of E-cadherin, highlighting its pivotal role in the adherent junction formation and occludin another protein involved in cell to cell adhesion contributing to the formation of tight junctions, which controls permeability and ion selectivity of paracellular pathway between adherent cells [18].

In addition, the reaction of cells and tissues to implanted foreign bodies depends on the material’s properties and its behavior upon contact with the body fluids. Räisänen et al. in 2000 Demonstrated that epithelial cells adhered and spread more avidly on metallic surfaces than on ceramic surfaces [19]. Moreover, pre-hemidesmosomes were found on metallic surfaces, but not on porcelain and aluminum oxide [19]. On the other hand, Säuberlich et al. in 1999 found a substantial cell adhesion to c.p. titanium, and a nonsignificant improvement when the surface was treated by sulphur dioxide plasma etching, plasma nitration orby silane coating [20].

Degidi et al. in a study performed in 2006 shown statistically significant differences with an overall lower inflammatory level in tissues surrounding zirconium oxide healing caps than at titanium caps [21]. Moreover, Mangano et al. in 2018 evaluated the expression of adhesion molecules in the soft tissues surrounding healing abutments produced by direct metal laser sintering technique, demonstrating that there was an increase in the intensity of expression of the integrins compared to machined screws [22]. It has been clearly shown that surface treatment and therefore different roughness can influence cell differentiation, protein film composition and molecules absorption [23]. Sinjari et al. in 2018 analyzed the fibrin clot extension on three different c.p. titanium topographies showing that the surface microtexture complexity determines the creation of a more extensive and three-dimensionally complex fibrin scaffold [23]. This could be of crucial importance for osseointegration and for the early formation of an effective connective tissue seal that would impair epithelial cells downgrowth. Recently, a new additive manufacturing technique, named Synthegra, has been introduced and extensively studied, offering a new technology for the treatment of titanium surfaces [23,24,25]. Among the laser conditioning techniques, Synthegra provides potential benefits in the field of implant dentistry and not only, because of its capability to directly build three-dimensional roughness with a pre-stabilized three-dimension topography. In fact, it presents pores with known dimensions: Diameter 5 microns; distance inter-pores 15 microns; and depth of the pore 5 microns [23,24,25]. Moreover, this laser treatment has been widely studied regarding bone responses and microbiological behavior. Specifically, concerning the proliferation of osteoblasts, it has been demonstrated that hemispherical porosity of 20-micron Laser-engineered generated could be responsible for cells proliferation and adhesion [25]. In addition, another study has verified the laser treatment effectiveness in relation to bacterial proliferation, demonstrating its significant efficiency in the reduction of the *P. gingivalis* biofilm formation [24].

Although the different efforts made, there are no ideal implants, healing screws or abutments surfaces available nowadays.

This is the first clinical study aiming to evaluate and compare by phenotypical and morphological analyses the performance in humans of laser-treated healing abutments compared to machined one.

The null hypothesis of this study was that no differences in terms of phenotypical and morphological analyses were present between the two different surfaces.

## 2. Experimental Section

### 2.1. Materials and Methods

#### 2.1.1. Study Design and Patient Selection

A total of 30 peri-implant soft tissues biopsies were gathered in order to perform this study. Immunohistochemical analysis of the soft tissues surrounding healing abutments (HAs) were performed. All samples come from the Operative unit of Implantology of the Department of Medical, Oral and Biotechnological Sciences, University “G.d’Annunzio”of Chieti-Pescara. All patients received information and instructions regarding the study protocol and each patient signed a written informed consent form. This clinical trial was approved by the “G.d’Annunzio” Chieti-Pescara University Ethic Committee on 11.07.2019 with reference *n*. 14. This study was carried out in accordance with the principles set out in the Helsinki Declaration developed by the World Medical Association on Humans.

The inclusion criteria were as follows:

- Patients between 18 and 75 years
- Patients with no systemic and/or oral health disease
- At least six months of healing after tooth extraction
- Adequate dimension of the attached gingiva (>2 mm) or keratinized tissue at the site selected and adequate residual bone crest

Meanwhile the exclusion criteria were:

- Patients with poor oral hygiene
- Plaque score (PS) and bleeding on probing (BOP) more than 25% [26]
- Patients with active periodontal disease
- Insufficient bone thickness for implant insertion and bone augmentation procedures
- patients who necessitate immediate implant loading protocols
- Uncontrolled diabetes mellitus
- Immune diseases
- Patients who smoke more than 10 cig/die

Then, 38 patients (mean age 56.5 ± 9.9 years), 13 women and 25 men were enrolled in the present study but only 30 patients were included for the immunohistochemical analysis as specified in Table 1. Eight patients were excluded because the keratinized tissue amount after healing was not sufficient, thus for not influencing the outcome of the implants we decided to do not perform the biopsy.

The study time points were organized as follows: T0 = first surgical stage: implant placement and recording of clinical data; T1 = second surgical stage after 12 ± 4 weeks recording of clinical data and insertion of experimental healing abutments; T2 (30 ± 7 days after T1) = biopsies for immunohistochemical analysis and experimental healing abutments collection and subsequently insertion of standard healing abutments. After the time point T2 all the prosthetic stages have been performed following the clinical good practice.

#### 2.1.2. Surgical Treatment

A complete hard and soft tissues inspection was performed and a panoramic radiograph or a Cone Beam Computed Tomography was taken as a first level survey for each patient. The implant insertion and surgeries were performed by an expert surgeon (M.P) in local anesthesia with 4% articaine solution containing 1:100,000 adrenaline. A crestal incision by performing a full-thickness flap and relative dissection were performed before the underlying bone profile was exposed. Then, the implant site was prepared with spiral drills of increasing diameter under abundant physiological irrigation, in order to prevent overheating of the bone as the manufacturer suggested. At the end of the surgical procedure, the implants were placed, showing a good primary stability; cover caps were screwed on and the fixtures were submerged. In fact, a two-stage implant placement surgery was performed for all the patients included. All implants used in the present study were (Omny, Geass s.r.l, Pozzuoli del Friuli, Udine, Italy) and had different diameters (3.50 mm, 4.1 mm) and lengths (7.0 mm, 8.0 mm, 10.0 mm, 11.5 mm). All patients were then subjected to oral antibiotic therapy with 2 gr per day for six days (Augmentin^®^, GLaxo-Smithkline Beecham, Brentford, UK). Detailed oral hygiene instructions as well as a 0.20% chlorhexidine rinse (Chlorexidine^®^, Oral-B, Boston, MA, USA) were also provided for seven days. Suture were removed after 7–10 days.

**Figure 1:**
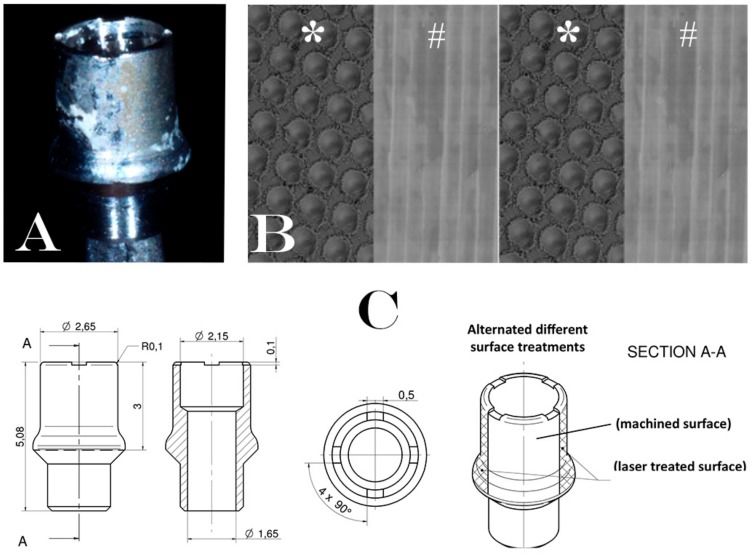
Description of experimental healing abutment used into the study: (**A**) Healing abutment after the removal of soft tissue. (**B**) Schematic representation of surfaces treatment on the healing abutment. Each healing abutment was treated with two alternated different surface treatments (laser treated (*)/machined (#)/laser treated (*)/machined (#)); (**C**) technical design of experimental healing abutment.

**Figure 2:**
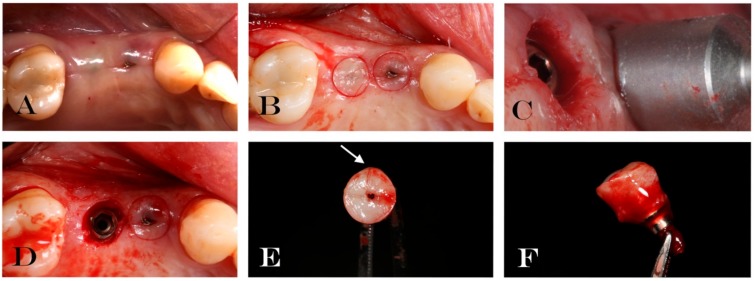
Explanatory case of treatment performed: (**A**) Soft tissue surrounding the experimental healing abutment; (**B**) circular incision drawing around the healing screw; (**C**) mucotome used for tissue biopsy; (**D**) implant after tissue removal; (**E**) occlusal vision of removal soft tissue and healing abutment. Arrow indicates micro-incision performed in order to know the spatial orientation of biopsy; (**F**) lateral vision of removal soft tissue and healing abutment.

After a healing period of 12 ± 4 weeks, during the second surgical phase, the implants were uncovered, the cover caps/screws were removed and replaced with a new experimental laser-treated/machined healing abutment (Geass s.r.l, Pozzuoli del Friuli, Udine, Italy). Each healing abutment was treated with two alternated different surface treatments (machined and laser treated surface) where the two surfaces were repeated with the following order: Laser treated/machined/laser treated/machined as shown in Figure 1. This order was performed to eliminate the bias of the different surface allocation. The HAs remained in situ for a period of 30 ± 7 days. In Figure 2 all the clinical phases have been described to better understand the procedures followed in this study.

#### 2.1.3. Specimen Retrieval and Analyses

After the healing period (30 ± 7 days), before starting with the impression taken the small diameter HAs both with the circular biopsies were retrieved for analysis as shown in Figure 2 and previously described [22]. The healing period was set at 30 days following a paper published by Femminella et al. in 2016 that described a complete re-epithelialization of the wound after 4 weeks [27]. The gingival biopsies were performed with a circular punch of 5 mm of diameter, as shown in Figure 2. The experimental HAs had a 2.65 mm of diameters leaving at least 1mm of soft tissue surrounded the healing screws. The samples obtained were processed for immunohistochemical analysis. The gingiva specimens were fixed in 10% neutral buffered formalin and then embedded to paraffin, identifying the point of passage between the region of the gingiva adherent to the laser-conditioned and the one adherent to the machined surfaces. Three µm sized paraffin embedded tissue sections, were deparaffinized, washed and blocked and then subjected to antigen retrieval, using 10 mM sodium citrate for 20 min at 60 °C.

The gingiva sections were stained with hematoxylin-eosin (Bio-Optica, Milan, Italy) or Trichrom (Bio-Optica, Milan, Italy) or May–Grünwald–Giemsa staining (Bio-Optica, Milan, Italy).

Immunohistochemistry was performed using the Mouse and Rabbit specific horseradish peroxidase/3,3’-Diaminobenzidine HRP/DAB (ABC) detection immunohistochemical IHC kit (Abcam Cambridge, UK) following manufacturer’s instructions. Briefly, samples were then incubated with anti-occludin antibody 5 µg/mL (Abcam Cambridge, UK) or human neutrophil defensins (alpha-defensins, HNP1–3) (Hycult Biotech, Uden, The Netherlands) for 1 h. Successively slides were incubated with goat anti polyvalent antibody for 10 min and subsequently with peroxidase for 10 min. After incubation samples were washed and treated by 3,3’-Diaminobenzidine DAB substrate. At the end of the process, slides were counterstained with hematoxylin-eosin [28].

Immunofluorescence staining was performed by blocking aspecificity and retrieving the antigen first and incubating then the section with the primary antibodies anti-E-cadherin 1:100 (Cell Signaling, Danvers, Massachussets, USA) or anti-β-catenin 1:100 (Cell Signaling Danvers, Massachussets, USA) or anti-K19 antibody 1:100 (Cell Signaling, Danvers, Massachussets, USA) overnight at 4 °C, as previously reported [29]. The samples were subsequently incubated with proper Alexa Fluor 488 goat anti-mouse or anti rabbit secondary antibodies for 45 min. Nuclei were counterstained with DAPI (VECTASHIELD^®^ Vibrance™, Vector Laboratories, Burlingame, CA, USA).

Histological observations were carried out using a Zeiss Axioskope light microscope (Zeiss, Jena, Germany) equipped with a Coolsnap Videocamera and the acquired images were analyzed with the MetaMorph 6.1 Software (Universal Imaging Corp, Downingtown, PA, USA).

Levels of primary antibody immunostaining were determined by analyzing the thresholded area percentage (CD63, HNP1–3, Occludin, E-cadherin, β-catenin) with the MethaMorph 6.1 program. Five randomly non-overlapping areas were chosen per each section, for the analysis as previously reported [30].

#### 2.1.4. Statistical Analysis

Statistical analysis was performed by analysis of variance (ANOVA test) using Origin 3.5 software for Windows (Microcal Software Inc., MA, USA) or by Kruskal-Wallis and Bonferroni-Dunn posthoc and Mann–Whitney tests using GraphPad Prism 4.0, as appropriate. All data are presented with mean ± standard deviation (SD); a value of *p* ≤ 0.01 was considered statistically significant.

## 3. Results

Thirty-eight patients were enrolled following the inclusion and exclusion criteria in the present clinical trial. Eight patients were excluded for the immunohistochemical analysis due to poor quality and quantity of keratinized tissue after first surgical stage. Thus only 30 soft tissue biopsies surrounding experimental healing abutments were performed and retrieved. All implants placed were osseointegrated, with no clinical signs of infection or marginal bone loss during second surgical stage. A survival rate of 100% at second surgical stage and no failures occurred during this study. Macroscopically, no signs of inflammation were present around the HAs and no clear differences between the laser treated and the machined surfaces were detectable as shown in Figure 2.

Moreover, PS and BOP around implants were recorded to be under 25%, as described in detail in Table 1. Clearly, microscopic analysis was necessary to evaluate the interface between the different surface treatments and the surrounding soft tissues.

Optical microscopic evaluation of these specimens revealed the area of the soft tissues adherent to the laser treated surface with a regular morphology in all its parts: The basal, prickle, granular, and keratinized layers of a healthy gingival epithelium were clearly distinguishable as well as the connective tissue of lamina propria and the basal lamina between the two tissues.

**Figure 3:**
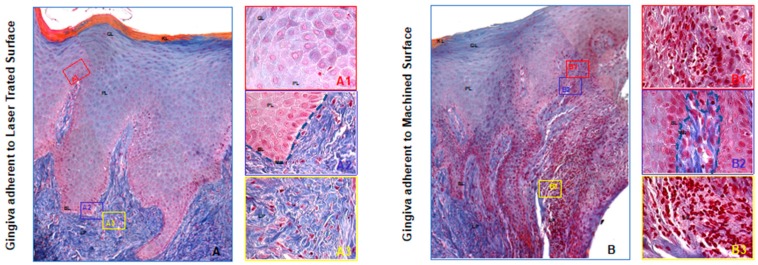
Trichrome staining of the region of the gingiva adherent to the laser-conditioned- (**A**) or to machined surfaces (**B**). In higher magnification of the gingiva adherent to laser treated surface it is evident the regular morphology in all layers: Junctional epithelium (**A1**), sulcular epithelium with all the typical layers including continuous basal membrane highlighted with the dotted blue line (**A2**) and subepithelial connective tissue of the lamina propria (**A3**). In higher magnification of the gingiva adherent to machined surface the typical morphology of the junctional epithelium is not respected (**B1**): the basal membrane is disrupted (highlighted with the dotted blue line in (**B2**) and the collagen fibers of subepithelial connective tissue of the lamina propria looks infiltrated with large inflamed area (**B3**). Magnification 10X (**A**,**B**) or 40X (**A1**–**3**,**B1**–**3**). Red, yellow and blue rectangles in A and B represent enlarged areas in the panels on the side, as indicated. Panel **A** and **B** are the results of sequential images mounted together. LP: Lamina propria; MB: basilar membrane (dotted blue line); BL: Basal Layer; PL: Prickle layer; GL: granular layer, KL: keratinized layer.

Whilst, the soft tissues area adherent to the machined surfaces showed several stresses features. Indeed, the basal membrane was disrupted and discontinued, moreover highly inflamed areas were detectable both in the epithelium and, in a larger extent, in the subepithelial connective tissue as demonstrated in Figure 3.

**Figure 4:**
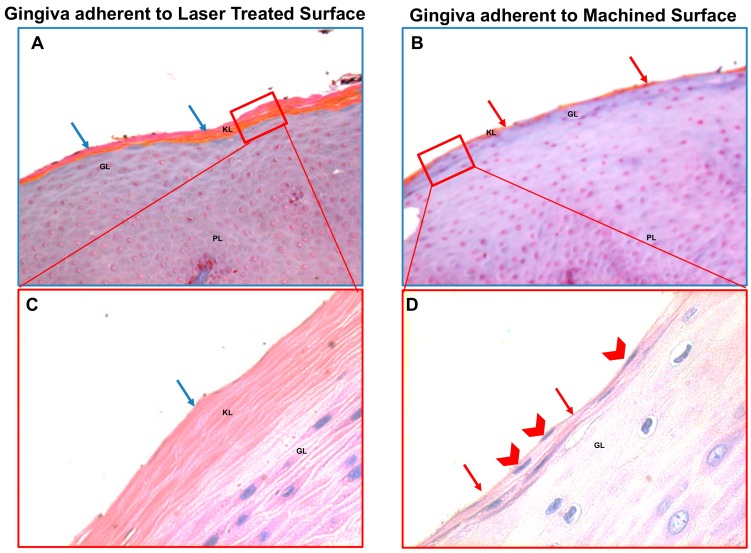
Trichrome staining (**A**,**B**) and May–Grünwald–Giemsa (**C**,**D**) staining of the region of the gingiva adherent to the laser treated (**A**,**C**) or to machined (**B**,**D**) surfaces, as indicated. The keratinized layer (that react in orange in the Trichrome staining and pink in the May–Grünwald–Giemsa staining) is barely present or absent (red arrows) in the gingiva adherent to the machined surface and the keratinized layer present still nucleated cells (red arrowheads) or a very thin enucleated cells layer compared to the regular corneal layer of the epithelium of the part of gingiva facing the laser-conditioned surface that present abundant anucleated keratinized layer (blu arrows). Magnification 20X (**A**,**B**) or 40X (**C**,**D**). Red rectangles in (**A**,**B**) represent enlarged areas in (**C**,**D**), as indicated PL: prickle layer; GL: granular layer, KL: keratinized layer.

**Figure 5:**
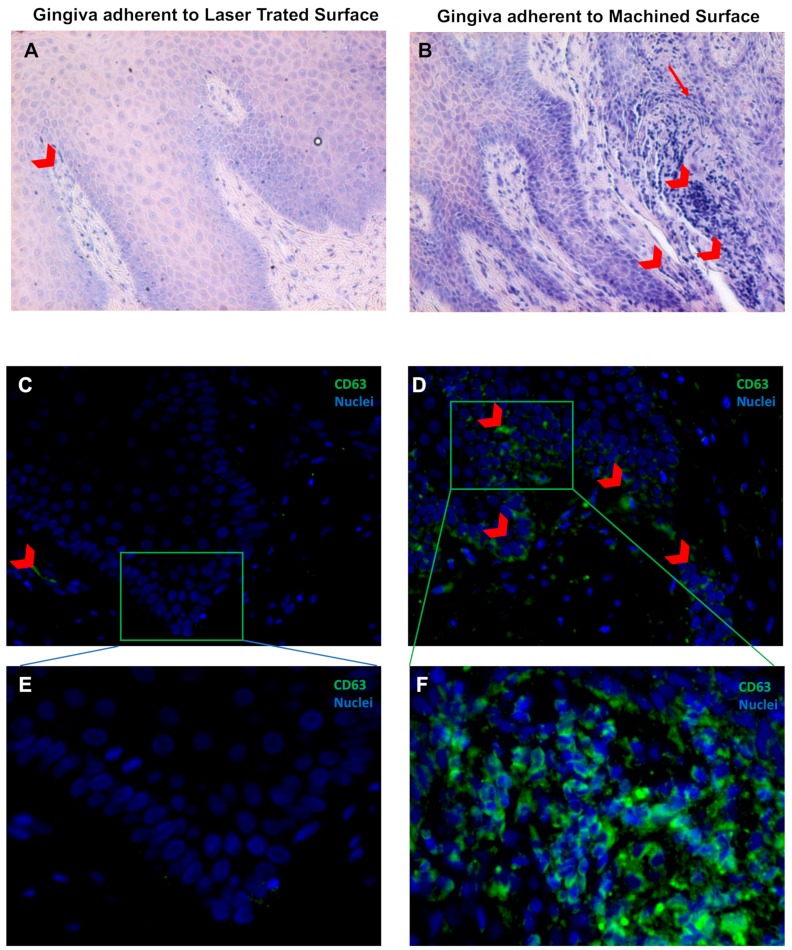
May–Grünwald–Giemsa staining (**A**,**B**) and Immunofluorescence against CD63 (green) (**C**,**D**) of the soft tissue area adherent to the laser-treated (**A**,**C**,**E**) or to machined (**B**,**D**,**E**) surfaces, as indicated. Red arrowheads indicate flogistic area with leukocites accumulation (**A**,**B**), that are also positive to CD63 immunostaining (**C**–**F**). Nuclei are counterstained with 4’,6-diamino-2-phenylindole DAPI in (**C**,**F**). Magnification 10X (**A**,**B**), 20X (**C**,**D**) or 40X (**E**,**F**).

In addition, the correct keratinization process is defective or absent in the region of the gingiva facing the machined surface since the keratinized layer present still nucleated cells or a very thin enucleated cells layer compared to the regular corneal layer of the epithelium of the part of gingiva facing the laser-treated surface (Figure 4). It was observed that the region of the gingiva adherent to the laser treated surface evidenced regular morphology and absence of flogistic area, while the continuity of the basal lamina of the epithelium is disrupted and associated with numerous area with leukocyte infiltration (Figure 5A,B). In fact, CD63 immunostaining showed positivity with a statistically increased inflammation both in the epithelium and in the connective tissue stroma of the gingiva adherent to machined surfaces (Figure 5C–E and Table 2).

**Figure 6:**
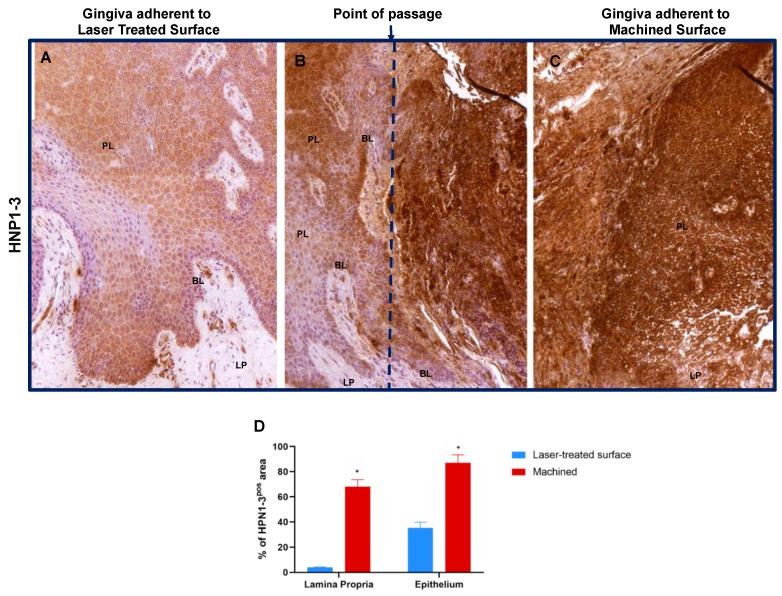
Immunostaining against HNP1–3 of the region of the gingiva adherent to the laser-treated (**A**), to the point of passage between the two surfaces (**B**) (dotted blue line represents an arbitrary hypothetical line) and to machined (**C**) surfaces, as indicated. Magnification 10X. (**D**) Percentage of HNP1–3 immunostaining positive area in the lamina propria and in the epithelial layers, as indicated. Values are expressed as mean ±SD of five determinations in randomly chosen sections for each specimen. * indicates values statistically different (*p* < 0.001) between region of the gingiva adherent to the laser-treated- or to machined surfaces, as indicated.

Moreover, HPN1–3 showed a positivity in the epithelium adherent to laser treated surface but not or lightly expressed in the lamina propria; otherwise it is dramatically more expressed in the gingiva faced to machined surface both in the epithelium and in the lamina propria, where it is also possible to appreciate an altered morphology of both the tissues. Moreover, in the junction between the soft tissue adherent to the laser and the one to the machined surfaces a gradual increase of HPN1–3 expression is detectable (Figure 6 and Table 2).

**Figure 7:**
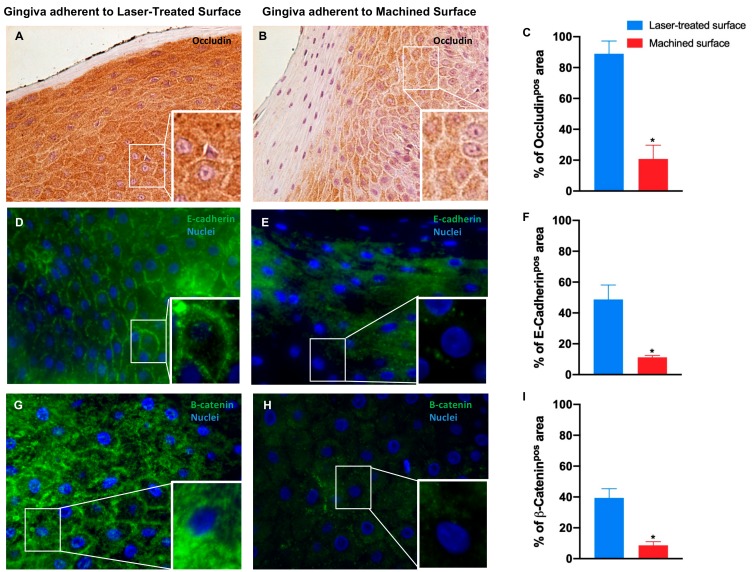
Immunostaining against occludin (**A**,**B**) and immunofluorescence for E-cadherin (**D**,**E**) and β-catenin (**G**,**H**) of the region of gingiva adherent to the laser-treated surface and to machined surface, as indicated. Magnification: 20X (or 40X in the inserts) in (**A**,**B**); 40X and (or 100X in the inserts) in (**D**,**E**,**G**). Percentage of occludin (**C**), E-cadherin (**F**) and β-catenin (**I**) positive staining area in the epithelial layers are pointed out in the bar graphs. Values are expressed as mean ±SD of five determinations in randomly chosen sections for each specimen. The (*) indicates values statistically different (*p* < 0.001) between the area of the soft tissue’s adherent to the laser-treated- or to machined surfaces, as indicated.

Immunohistochemical analysis showed that in the gingiva epithelium adherent to machined surface, Occludin is strongly downregulated, meaning that tight junctions are altered and less represented, while it is highly expressed in the gingival epithelium facing the laser treated surface, suggesting a regular morphology and functionality (Figure 7A–C and Table 2).

Immunofluorescence staining revealed that also E-cadherin expression is significantly reduced in the epithelium facing the machined surface compared to the gingiva epithelium facing the laser conditioned epithelium. It is also notable the disruption of the typical organization in the first one in opposition to the correct localization of the protein on the cell membrane in the second one (Figure 7D–F and Table 2).

Furthermore, β-catenin is almost undetectable in the epithelium facing the machined surface but it is regularly expressed and localized in the laser-treated surface epithelium (Figure 7G–I and Table 2).

## 4. Discussion

This study performed an immunohistochemical analysis of the soft tissues surrounding different treated healing abutments surfaces. The present results demonstrated how the soft tissues around implant healing abutments respond, at the same implant site, differently to different surfaces (laser treated vs. machined one). These results probably will open new research fronts being the first one on this type of laser treated surface on humans. Moreover, it seems that this kind of surface has good chances to influence positively the challenging coronal biological seal formation. To date, there are no available solutions which can inhibit the marginal bone loss or the peri-implantitis due to the microgap and the bacterial colonization [31,32]. Thus, prevention of downgrowth epithelial cells and microbial load at the fixture-abutment interface is one of the major challenges in the development of two-piece implant systems [11,33].

Novel materials and surface modifications are being continuously developed to improve clinical performance of dental abutments and implants.

Kasten et al. (1990) found higher epithelial cell adhesion on hydroxyapatite compared with c.p. titanium, but the extremely low number of samples limits the significance of their results [34]. Moreover, human gingival fibroblasts attachment to c.p. titanium proved to be significantly higher than to porous and non-porous hydroxyapatite [35]. Mangano et al. in 2018 shown that the degree of cell adhesion and the inflammatory infiltrate of the surrounding soft tissues is influenced by the HAs surface [22]. In this study, however, patients had a healing abutment per surface tested and not all the surface types simultaneously. In these results, it was possible to appreciate the different response of the different screws types in contiguous sites, demonstrating how soft tissues healing could be actually influenced by the surface treatment.

To date, the literature has not provided univocal answers on which is the best way to condition the peri-implant tissues [36]. Some authors have shown how a smooth surface can reduce plaque retention and bacterial adhesion, consequently reducing the risk of inflammation [37,38]. Often, however, these results have been achieved in animal model studies [37,38] and not in human one. On the contrary, other authors concluded that treated surfaces can positively influence the soft tissue healing [22,39].

Over the years, different authors have studied this subject [40,41]. Dellavia et al. in 2013 [40] studied the inflammatory response in peri-implant soft tissues in implant supported restoration realized with switched platform abutments. The immunohistochemical analysis concluded that there were no statistically significant differences between switching and non-switching platforms [40]. In another paper [41] they also analyzed the marginal bone loss (MBL) with the same prosthetic configurations of the previous study. The authors showed that platform switching had a positive influence on MBL but again no difference in terms of inflammatory infiltrate on soft tissues were found [41].

Among the causes of bone resorption, the frequent screwing and unscrewing of secondary components has been shown to have a negative role. However, in this study the healing abutments were removed within a limited number of times (3 times) in order to limit the damage of soft tissues. In addition, recent studies have shown that clinically this can have an extremely limited effect and other factors may have a greater influence [42,43]. Moreover, during the healing period the test HAs used in this study have not been removed. Moreover, the use of alternative materials has provided expectations for improving the coronal seal of peri-implant soft tissues [13,21]. Specifically, zirconia healing screws have been also used. In fact, Degidi et al. in 2006 [21] conducted a comparative analysis demonstrating how a lower inflammatory level was present around zirconia screws. On the other hand, however, there are no long-term follow-ups on the use of these screws [13]. Differently, at the moment titanium represents the only material that can be used with long term satisfactory results at the interface with soft tissues [13].

The modification of the surface topography has shown different results, demonstrating in any case an impact on the early events of soft tissue healing. Some treatments have shown an improvement in adhesion [22] others a reduction of epithelial adhesion [44,45] and others a cell growth that occurred indifferently between the two surfaces [46,47].

On the other hand, some preclinical or animal studies have shown that a laser surface treatment can positively influence soft tissue seal around implants [48,49]. Our results, demonstrated a positive influence of the surface alterations in terms of surface topography, in a human study with experimental healing abutments that provided testing and control surfaces simultaneously at the same implant site. In this regard, these data suggest that the machined surface might have a role in the inflammatory process of the tissues with which is in contact, while the laser-treated seems to be better tolerated and do not induce any inflammation.

Moreover, it was also evaluated, with the mean to better understand the behavior of peri-implant soft tissues, the integrity and the functionality of the gingiva epithelium by studying intracellular adhesion molecules such as occludin, E-cadherin, and β-catenin. In fact, it was demonstrated that the laser treated, but not the machined surface, maintains the integrity and functionality of the gingiva epithelium.

Specifically, occludin is commonly used to identify tight junctions, that controls the paracellular passage creating a regulable semi permeable diffusion barrier between individual cells. Thus, when the tight junction integrity is affected, periodontitis infections might find a better homing situation in case [15]; E-cadherin is a desmosome marker and its proper expression is essential to maintain the integrity of the epithelium [50], as well as β-catenin, a protein that localizes in the adherents junctions [51]. In the present study, different β-catenin expression suggested that the machined surface induce impaired cell to cell interaction, adversely affecting proper expression of both tight and adherent junctions, as well as desmosomes, while the laser-treated one do not influence the right organization of the junctional epithelium. Since Trichrome staining showed area that resembled inflamed, the inflammation by May–Grünwald–Giemsa staining (which is routinely used to distinguish blood cells) and by immunofluorescence for CD63 [52], showed regular morphology in the gengiva adherent to the laser treated surface, meanwhile the continuity of the basal lamina of the epithelium is disrupted and associated with numerous area with leucocytes infiltration in the gingiva adherent to machined surfaces.

In addition, HNP-1–3, taken into consideration in this study, belongs to these defensins family present in the clinically healthy junctional epithelium and in the pocket epithelium of gingiva with periodontitis, being chemoattractant for monocytes and T-cells. It has been demonstrated that, high tissue concentrations of HNPs during periodontal disease may affect the epithelial cells and fibroblasts [14]. In few studies, low to moderate expression of HNP1 was found to be mitogenic increasing the cell proliferation and migration and wound closure [14,53]. High concentrations of HNPs (>10 mg/mL), however, promote apoptosis of epithelial cells and, more interestingly, stimulates the bacterial adherence to epithelial cells [14]. It is well known that the junctional epithelium is a barrier against oral bacteria with a continuing neutrophil influx. Interruption of the epithelium structure not only impairs the defense against periodontopathogenic bacteria but may also give the chance tobacteria, such as *Fuso-bacterium nucleatum* and *Prevotella intermedia*, to invade host tissues [54]. Thus, it was hypothesized that HNP1–3 molecules contribute to epithelial homeostasis in healthy conditions by increasing mitogenesis in epithelial cells and their attachment to hard surfaces. On the other hand, in presence of inflammation, defensins concentration dramatically increases exhibiting adverse effects by increasing the cell death and bacterial attachment to the epithelium.

In fact, this study demonstrated that the a-defensins were expressed in all the specimens but with a higher expression on the laser-treated surface rather than on the machined one. Moreover, on the latter it can be observed a loss of the typical tissue morphology. It is also evident, the increased expression intensity of this marker on the passage interface between laser treated and machined surfaces.

The molecules identified and analyzed provided a clear answer demonstrating a really different behavior with respect to the type of surface morphology. Clearly, these results should be replicated applying the same study design in a longer follow-up period to better understand the biological behavior of the soft tissues regarding these two different surfaces. Taken into consideration that the laser-treated surface morphology has provided clear biocompatibility results in relation to the growth of osteoblasts [25], the reduced bacterial proliferation [24] and the formation of a stable clot [23] we can assume that it encourages the use of laser-treated surface on healing abutments.

Although, the results of the study provide information about a limited period of time (30 days) of soft tissue healing they encourage also the use of this surface in the transmucosal portion. However, it is important to remember that this is the first in vivo study performed in this type of surface treatment. Thus, the authors strongly recommend further future studies not only in a longer period but also in presence of peri-implant pathologies to verify its goodness in different situations. In fact, one of the major limitations of the present study is that we did not perform a microbiological evaluation, to better understand if the inflammation present was induced by bacteria. Thus, we strongly recommend an immunohistochemical and microbiological study, maintaining the same study design as the present but with a longer follow up period.

## 5. Conclusions

These data suggest that the use of laser treated surface on healing abutment could positively influence the healing of peri-implant soft tissues and it is preferable to maintain the integrity and functionality of the gingiva epithelium. The positive influence in cell adhesion and the reduced inflammatory infiltrate of the peri implant soft tissues on the laser treated surface, suggests its clinical use. However, further studies will be necessary to better understand biological aspects related to soft tissue adhesion not only on abutment surfaces but also on prosthetic materials.

## Figures and Tables

**Table 1:** Demographical and clinical parameter of the patient enrolled.

Subject Characteristics	*n* = 38
Age Mean	56.55 ± 9.95 years
Gender (Male)	25 (65.79%)
Implant Position (Upper)	25 (65.79%)
Implant Position (Lower)	13 (34.21%)
Final Torque Insertion (NCM) Mean	49.73 ± 11.05
BOP# Mean at Implant Placement	7.32%
BOP Mean at Second Surgical Stage	9.68%
PS # Mean at Implant placement	13.87%
PS Mean at Second Surgical Stage	17.76%
Implant Success at Second Surgical Stage	38 (100%)
Biopsy Performed and Analyzed	30 (78.94%)

#Plaque Score (PS) Bleeding on probing (BOP).

**Table 2:** Expression pattern of inflammation and junctional markers. Statistically significant differences between different surface treatment were found (*p* < 0.001).

Antibody	Epithelium Facing the -		Lamina Propria Facing the -	
	Laser Treated Surface	Machined Surface	Laser treated surface	Machined Surface
CD63#	2.6 ± 1.2	9.3 ± 1.6 *	6.1 ± 3.2	30.3 ± 12.5 *
HNP1–3#	34.9 ± 4.7	87.0 ± 6.6 *	4.1 ± 0.4	68.0 ± 5.4 *
Occludin	88.9 ± 8.2	20.8 ± 8.9 *	-	-
E-Cadherin	48.7 ± 9.5	11.2 ± 1.2 *	-	-
β-Catenin	39.4 ± 6.0	8.6 ± 2.5 *	-	-

Values indicate the percentage of positive area * indicates significant statistically difference (*p* < 0.001) between region of the gingiva adherent to the laser-treated- and to machined surfaces. #Cluster of differentiation (CD63) - #human neutrophil defensing (HPN1–3).
